# Case report: Spontaneous bilateral intraocular lens dislocation in a patient with homocystinuria

**DOI:** 10.3389/fcvm.2022.974842

**Published:** 2022-09-14

**Authors:** Bangtao Yao, Xujian Chen, Gang Liu, Xiaogui Zhao

**Affiliations:** ^1^Department of Ophthalmology, Nanjing Lishui People’s Hospital, Zhongda Hospital Lishui Branch, Southeast University, Nanjing, China; ^2^Department of Ophthalmology, Nanjing Lishui District Hospital of Traditional Chinese Medicine, Nanjing, China

**Keywords:** ectopia lentis, homocystinuria, spontaneous, high myopia, lamellar macular hole, osteoporosis, CT

## Abstract

**Background:**

Spontaneous bilateral intraocular lens dislocation of the vitreous cavity is a rare ocular disorder. This article aims to comprehensively describe bilateral spontaneous intraocular lens dislocation with unilateral lamellar macular hole and retinoschisis in a Chinese woman with homocystinuria.

**Case presentation:**

A 72-year-old Chinese woman with homocystinuria presented with a painless bilateral blurring of vision. The slit lamp showed the absence of lenses in both eyes. B-ultrasound and orbital computed tomography (CT) demonstrated bilateral posterior dislocation of the crystalline lenses, and spectral-domain optical coherence tomography (SD-OCT) revealed a lamellar macular hole and retinoschisis in the right eye. Biochemical examination demonstrated that the total homocysteine level was moderately elevated.

**Conclusion:**

This report is the first to present an extensive and valuable description of bilateral intraocular lens dislocation with unilateral lamellar macular hole and retinoschisis secondary to homocystinuria. We have demonstrated that this case was spontaneous and chronic. CT is an effective diagnostic tool for patients with ectopia lentis. Early diagnosis and suitable management of patients with homocystinuria are essential to prevent these complications.

## Introduction

Homocystinuria is a rare autosomal recessive disease mainly caused by the absence of cystathionine-β-synthase, which is involved in the methionine metabolism pathway, affecting 0.82 in 100,000 the population worldwide (1, 2). Untreated cases result in multisystemic disorders, including ocular diseases, skeletal abnormalities, developmental delay, and thromboembolic events (3).

Ectopia lentis, also known as lens subluxation or dislocation, is the most significant ocular complication in patients with homocystinuria (4). Previous reports have documented that cystathionine-β-synthase deficiency could influence the nutritional metabolism of the lens zonule, leading to their degeneration and disruption (5). A completely dislocated lens can move freely from the original position into the anterior chamber or vitreous cavity, which may result in acute angle-closure glaucoma and uveitis (1, 6).

To our knowledge, complete lens dislocation into the vitreous cavity associated with a lamellar macular hole and retinoschisis in homocystinuria has not been reported.

## Case description

A 72-year-old Chinese woman with homocystinuria presented with a bilateral painless blurring of vision for several decades. She said that the problem had occurred since childhood; however, it had remained untreated. The patient did not complain of metamorphopsia or recent vision deterioration. The patient did not also complain of any other systemic symptoms, such as chest tightness, palpitation, dizziness, or headache. The patient had no history of trauma or surgery, high blood pressure, diabetes mellitus, hyperlipidemia, coronary atherosclerotic heart disease, Marfan syndrome, or Marchesani syndrome. The patient’s parents were close relatives.

**FIGURE 1:**
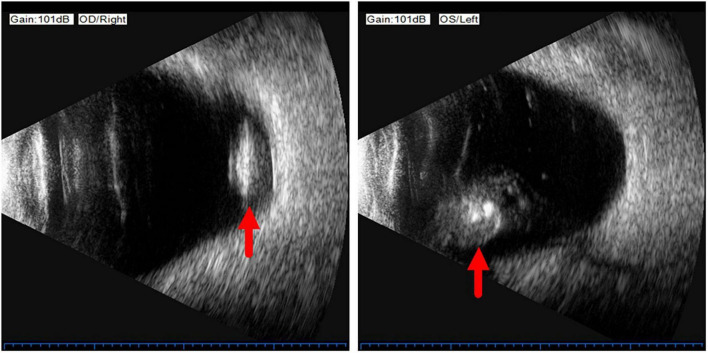
B-ultrasound showed that the lenses were located in the vitreous cavity in both eyes (red arrows).

**FIGURE 2:**
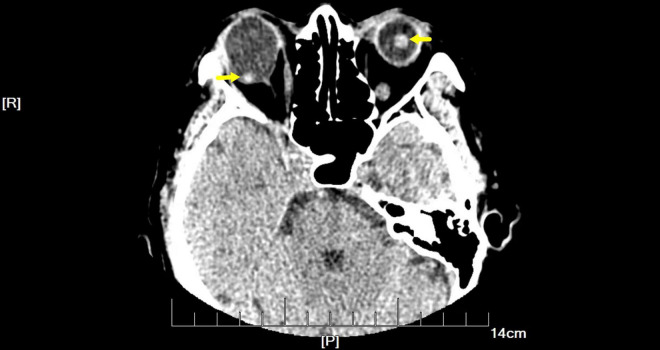
Orbital computed tomography (CT) demonstrated free-floating lenses in the posterior poles (yellow arrows).

**FIGURE 3:**
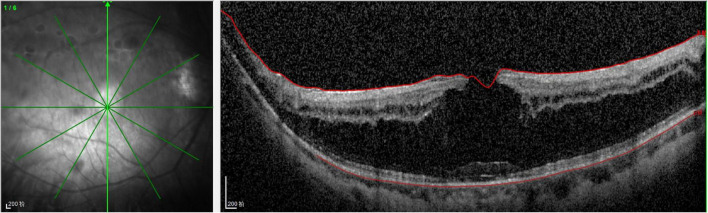
Spectral-domain optical coherence tomography (SD-OCT) revealed the lamellar macular hole and retinoschisis in her right eye.

On examination, her height and weight were 165 cm and 66.2 kg, respectively. The best-corrected visual acuity was 20/300 OD and 20/250 OS, with a refractive status of +5.25/−2.75 × 25 OD and +5.75/−3.25 × 154 OS. The anterior segment showed the absence of lenses in both eyes. Fundus examination revealed optic atrophy, staphyloma, and chorioretinal atrophy. The initial intraocular pressure was 16 mm Hg OD and 17 mm Hg OS, and the axial length was 31.11 mm OD and 29.33 mm OS. B-ultrasound showed that the lenses were located in the vitreous cavity in both eyes (red arrows, Figure 1). Orbital computed tomography (CT) demonstrated free-floating lenses in the posterior poles (yellow arrows, Figure 2), and spectral-domain optical coherence tomography (SD-OCT) revealed a lamellar macular hole and retinoschisis in the right eye (Figure 3).

The plasma total homocysteine concentration was 49.1 μmol/L (normal 0–15). The X-ray bone density test showed osteoporosis (−2.9 g/cm^2^), and a slender appearance with arachnodactyly was not observed. All other investigations were normal.

The patient was diagnosed with homocystinuria, osteoporosis, ectopia lentis, lamellar macular hole, and retinoschisis. The patient was treated with vitamin B6 and folic acid. She chose to defer surgery and was advised to undergo physical and ophthalmic examinations every 6 months. During the 3-year follow period, her condition remained stable. The patient did not provide any other chief complaints. Her intraocular pressure was normal, and SD-OCT images remained unchanged.

## Discussion

Homocystinuria is a rare autosomal recessive metabolic disease caused by cystathionine-β-synthase deficiency (1). It is diagnosed by elevated plasma total homocysteine concentration (7). Clinically, it is characterized by ocular disorders, skeletal abnormalities, developmental delays, and thromboembolic events (3).

Ocular complications secondary to homocystinuria include ectopic lentis, high myopia, secondary pupillary block glaucoma, optic atrophy, and retinal detachment. Ectopia lentis (lens subluxation or dislocation) is the most significant manifestation of these complications and was first described by Dr. Karl Stellwag in 1856 (1, 4).

Ectopia lentis is typically inferonasal and has been observed in more than 90% of untreated patients with homocysteinemia in their third decade of life (1). Age has been reported as a predictor of zonular instability and disruption, and complete dislocation is frequently observed in older patients (1). The lens can move significantly either into the anterior chamber or the vitreous cavity with or without angle-closure glaucoma. However, bilateral posterior lens luxation is rare.

The biochemical mechanism of ectopia lentis in homocystinuria is not yet fully understood. Defects in fibril disulfide bridges may provide a biochemical basis for lens dislocation (5). Previous reports have shown that the deficiency of cystathionine-β-synthase could influence the nutritional metabolism of the lens zonule, which causes their degeneration and rupture. Furthermore, elevated homocysteine levels may interfere with the cross-linking of sulfhydryl groups in elastin (8).

Myopia is secondary to lens instability and axial elongation in patients with homocysteinemia. Previous reports have documented that high myopia is related to pathological changes in weak scleral connective tissue, and it was present in almost 45% of homocystinuria cases (4). Hsia et al. suggested that a lamellar macular hole in association with retinoschisis is specific to myopic tractional maculopathy (9). In the present case, a lamellar macular hole and retinoschisis secondary to high myopia were observed on SD-OCT images. A lamellar macular hole is a common macular structural defect in high myopia tractional maculopathy; however, its formation is complicated. Lamellar hole-associated epiretinal proliferation and posterior vitreous adhesion occur more frequently in eyes with high myopia (10).

In the present case, homocystinuria presented with ophthalmic manifestations of osteoporosis. Other complications, such as developmental delay and thromboembolism, were not present, possibly due to the well-controlled homocysteine concentration for decades.

Bilateral ectopia lentis is closely associated with several systemic disorders (6). Therefore, differential diagnoses, such as Marfan syndrome, should be carefully addressed. Patients with Marfan syndrome typically have arachnodactyly and a tall slender appearance, and ectopia lentis is commonly subluxated and superior temporal (11).

Clinically, vitamin B6 and folic acid have proven effective for homocystinuria, reducing the incidence of complications (1). However, the treatment of ectopia lentis remains controversial. Surgical intervention in patients with anterior lens dislocation with or without secondary acute glaucoma is recommended. Surgical decisions must be made with caution in cases, where the lens is dislocated into the vitreous cavity, as postoperative retinal detachment and vitreal prolapse have been reported (12). In the present case, the patient chose to defer surgery and was treated with vitamin B6 and folic acid. During the 3-year follow-up period, her condition remained stable.

However, the present study has some limitations. First, the sample size of this study was small. Second, the enzyme activity assay was not performed.

In conclusion, we described the first case of bilateral intraocular lens dislocation with a lamellar macular hole and retinoschisis in a patient with homocystinuria. We have demonstrated that this case was spontaneous and chronic. Neonatal screening and early diagnosis are essential for preventing these complications.

## Data availability statement

The original contributions presented in this study are included in the article/Supplementary material, further inquiries can be directed to the corresponding authors.

## Ethics statement

The studies involving human participants were reviewed and approved by the Nanjing Lishui People’s Hospital. The patient/participant provided their written informed consent to participate in this study and for the publication of this case report.

## Author contributions

BY wrote the manuscript, established the diagnosis, and reviewed the manuscript. XC dealt with the figures and consulted the literature. GL edited the manuscript. XZ reviewed the manuscript. All authors have read and approved the final manuscript.
