# Supplementary figures and images for: Case report: Spontaneous bilateral intraocular lens dislocation in a patient with homocystinuria
Source: Front Cardiovasc Med. 2022 Sep 14;9:974842. doi: 10.3389/fcvm.2022.974842 (PMC9515901; doi:10.3389/fcvm.2022.974842)

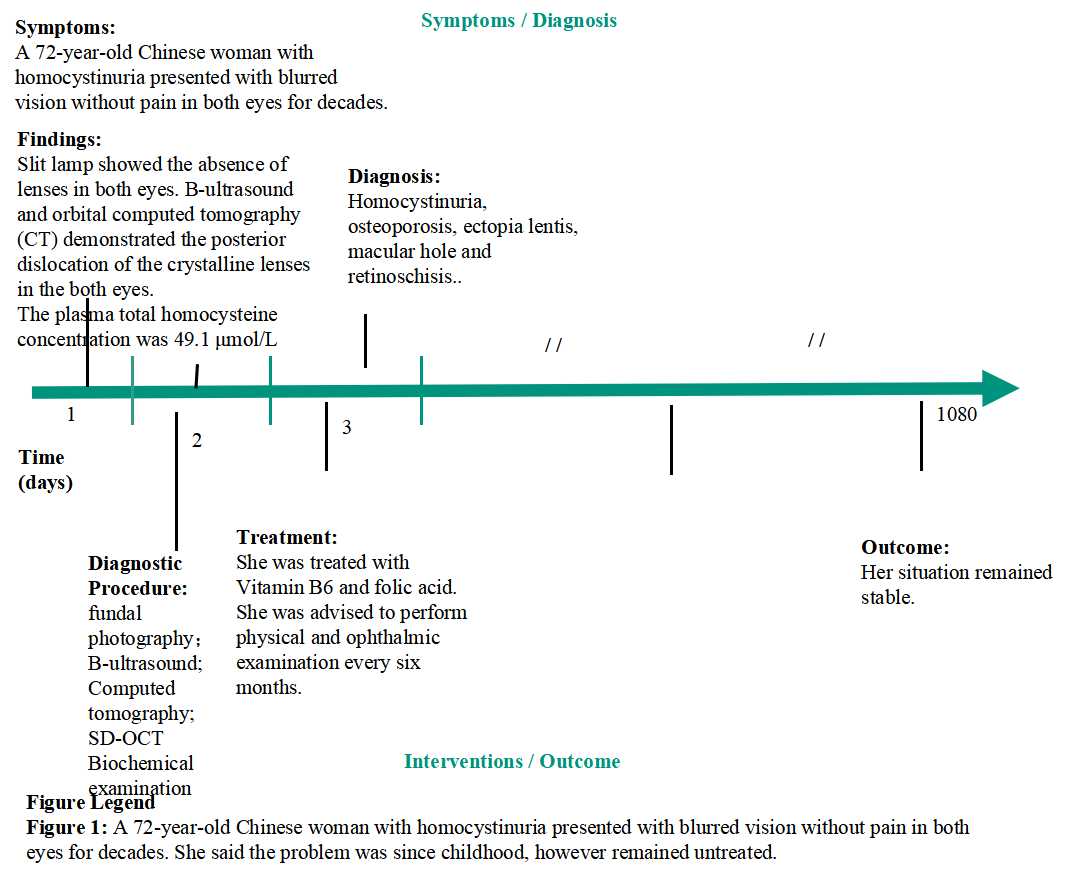

Supplement: Supplementary file 1 [file Image_1.JPEG]
